# Risk factors for previously undiagnosed primary open-angle glaucoma: the EPIC-Norfolk Eye Study

**DOI:** 10.1136/bjophthalmol-2020-317718

**Published:** 2021-06-25

**Authors:** Michelle P Y Chan, Anthony P Khawaja, David C Broadway, Jennifer Yip, Robert Luben, Shabina Hayat, Tunde Peto, Kay-Tee Khaw, Paul J Foster

**Affiliations:** 1 UCL Institute of Ophthalmology, UCL, London, UK; 2 NIHR Moorfields Biomedical Research Centre & UCL Institute of Ophthalmology, London, UK; 3 MRC Epidemiology Unit, University of Cambridge, Cambridge, UK; 4 Department Ophthalmology, Norfolk & Norwich University Hospital, Norwich, UK; 5 Clinical Research Department, International Centre for Evidence on Disability, London, UK; 6 Department of Public Health and Primary Care, University of Cambridge, Cambridge, UK; 7 Centre for Public Health, Blackwell's Queen's University Belfast, Belfast, UK

**Keywords:** glaucoma, epidemiology

## Abstract

**Background and aim:**

Undiagnosed glaucoma is an invisible but important public health issue. At least half of glaucoma cases are estimated to be undiagnosed in western populations. The aim of this study is to examine risk factors for previously undiagnosed primary open-angle glaucoma (POAG).

**Design:**

Cross-sectional study within the European Prospective Investigation of Cancer-Norfolk Eye Study, a large-scale cohort study in the UK.

**Participants:**

314 study participants with POAG in either eye.

**Methods:**

Logistic regression was used to examine associations with previously undiagnosed POAG compared with previously diagnosed POAG. The factors examined included sociodemographic, ocular, physical and economic factors that could be barriers to eye care access.

**Results:**

217 participants had previously diagnosed POAG and 107 participants were newly diagnosed with POAG during the study. After adjusting for covariables, the factors significantly associated with previously undiagnosed POAG were: a lower pretreatment intraocular pressure (IOP) (OR 0.71/mm Hg, 95% CI 0.63 to 0.80, p<0.0001), and to have reported no problems with their eyesight (OR 0.03, 95% CI 0.01 to 0.10, p<0.0001).

**Conclusions:**

The risk factors for previously undiagnosed POAG identified in this study highlight the over-reliance on IOP level in glaucoma screening and the risk of missing glaucoma among lower IOP cases. It also suggests a role in improving glaucoma awareness in the community.

## Introduction

Undiagnosed glaucoma is an invisible yet sizeable public health issue. Eye surveys conducted in developed western countries have consistently shown that at least half of glaucoma cases are previously undiagnosed.^1 2^ Published studies have described the clinical features and risk factors of undiagnosed glaucoma compared with previously known cases,^3–5^ which include lower education attainment,^3^ not having seen an ophthalmologist in the prior year^4 5^ or seeing an optometrist rather than an ophthalmologist.^3 5^ There are also reasons to suspect that the clinical features and severity of glaucoma may differ between previously diagnosed and patients with undiagnosed glaucoma. The features shown to be significant for undiagnosed glaucoma are: smaller vertical cup to disc ratio, a negative family history of glaucoma,^4^ presence of visual field detect,^5^ lower mean baseline intraocular pressure (IOP) and baseline hyperopia.^3^ The aim of this study was to examine the associations with previously undiagnosed primary open-angle glaucoma (POAG) in the European Prospective Investigation of Cancer (EPIC)-Norfolk Eye Study, a large British community cohort, focusing on the potential roles of ocular features, physical factors and social barriers to eye care access in undiagnosed POAG.

## Methods

### Study design

The EPIC study is a pan-European multi-cohort study, designed to investigate the lifestyle determinants of cancer risks. The EPIC-Norfolk cohort was established in the city of Norwich and the surrounding rural and urban areas, in the eastern English county of Norfolk, between 1993 and 1997.^6^ A total of 30 445 men and women aged 40–79 years were recruited at a baseline survey from the databases of 35 general practices. The predominant ethnicity of the cohort was white, and included individuals across the range of socioeconomic status and educational achievements. The EPIC-Norfolk Eye Study was carried out between 2004 and 2011 when ophthalmic data were collected from 8623 participants.^7^

Detailed descriptions of the study design^7^ and glaucoma diagnosis^8^ of the EPIC-Norfolk Eye Study have been reported previously. In brief, all subjects were examined systematically in a screening test, which included tonometry (ORA; Reichert Corporation, Philadelphia, USA), optic nerve head assessment (fundus photos and Heidelberg Retina Tomograph II) and peripapillary nerve fibre layer assessment with scanning laser polarimetry (GDx-VCC, Zeiss, Dublin, California, USA). A 24-2 central threshold visual field test (Humphrey 750i Visual Field Analyzer, Carl Zeiss Meditech, Welwyn Garden City, UK) was performed in participants with abnormal findings suspicious of glaucoma on HRT or GDx-VCC, and in 1 out of 10 subjects with normal findings. Subjects with abnormal findings who met a set of predefined criteria designed to detect glaucoma were referred to the Eye Department of the Norfolk and Norwich University Hospital for a definitive eye examination by a consultant ophthalmologist with a specialist interest in glaucoma (DCB). Full details of these criteria were published previously.^7^ Glaucoma was defined as the presence of structural optic disc abnormalities and visual field loss, with no other explanations for the disc and field appearances. POAG is defined as glaucoma in the presence of open anterior angles with no known secondary causes. The differentiation of high tension glaucoma (HTG) and normal tension glaucoma (NTG) was based on IOP level before glaucoma treatment commenced. HTG was defined as untreated IOP >24 mm Hg on one occasion, or >21 mm Hg on at least two separate occasions or on diurnal IOP phasing. NTG was defined as untreated IOP ≤21 mm Hg on at least two separate occasions or on diurnal IOP phasing. An individual participant’s glaucoma diagnosis was defined by taking the clinically more serious diagnosis of either eye, in the following hierarchy (most serious to least serious): glaucoma, glaucoma suspect, ocular hypertension, narrow angle spectrum (primary angle closure, primary angle closure suspect and narrow angles) and normal. Self-reported data including family history of glaucoma, contact lens or glasses wear, eyesight problem and health status were all collected from a self-administered questionnaire.

### Statistical analysis

Logistic regression was used to analyse the risk factors for previously undiagnosed POAG, with the dependent variable coded as 0=subjects with known POAG and 1=subjects with previously undiagnosed POAG. Factors that were significant (p<0.05) in the univariable analysis were included in the multivariable model in a stepwise backward model, and removed if p<0.05. The factors examined (box 1) included socioeconomic, demographic and systemic factors, as well as ocular factors that could affect a subject’s likelihood of seeking eye care, such as presence of low visual acuity, previous cataract surgery, self-reported eyesight problems, high refractive error or wearing glasses/contact lens. Physical and economic factors that could present a barrier to eye care access, such as financial difficulty, physical frailty and poor health status were also included. Cup/disc ratio (CDR) and CDR asymmetry were multiplied by 10 in the regression models to allow the OR to be analysed per 0.1 increase in CDR. For ocular factors, the worse value of the two eyes were used for visual acuity, axial length (longer value) and central corneal thickness (lower value). For IOP, CDR and visual fields mean deviation, the value of the OAG eye was used; if both eyes had OAG then the worse value was used. All statistical analyses were performed using STATA (Stata/SE V.13.1).

Box 1 **List of potential risk factors examined in the study**

**Sociodemographic factors**

- Age, sex.
- Social class by occupation.
- Highest educational qualification.
- Employment status.
- Self-reported financial status.

**Systemic factors**

- Systolic blood pressure.
- Diastolic blood pressure.
- Body mass index.
- Diabetes status.

**Ocular risk factors**

- Glaucoma type.
- Goldmann-correlated IOP of the glaucomatous eye.
- Higher cup/disc ratio (CDR) on disc photos of either eye.
- CDR asymmetry.
- Axial length.
- Visual field (mean deviation).
- Central corneal thickness.

**Proxy factors for eye care seeking behaviour**

- Self-reported family history of glaucoma (in any blood relation).
- Self-reported glasses/ contact lens wear (yes/no).
- Self-reported eyesight problem (yes/no/don’t know).
- Previous cataract surgery in either eye.
- Maximum absolute refractive error of either eye.
- Worse visual acuity of either eye.
- Self-reported health status (excellent or very good/ good/fair/ poor).

### Pretreatment IOP and its imputation

To allow unbiased comparison of IOP levels between the two groups, the pretreatment IOP was used for participants who have had pressure-lowering treatment. Pretreatment IOP is defined as the highest IOP (Goldmann applanation tonometry) documented in the patient’s hospital records before any IOP-lowering treatment (drops or surgery) was instigated. For those who have had IOP-lowering treatment but the pretreatment IOP was unavailable, the pretreatment IOP was imputed. Imputed IOP was not used in the diagnosis of NTG and HTG, which was made based on measured untreated IOP.

Among the 314 POAG participants in the study, 213 HTG eyes and 100 NTG eyes were on pressure-lowering treatment, defined as being on pressure-lowering medication and/or having undergone glaucoma surgery. In 114 HTG eyes and 42 NTG eyes the pretreatment IOP was available. Due to the high proportion of missing values (missing at random), they were imputed using multiple imputation by linear regression. The predictors were age, sex and OAG type (NTG vs HTG), and 100 iterations were imputed.

## Results

Out of the 8623 participants in the EPIC-Norfolk Eye Study, 363 participants were diagnosed with glaucoma in either eye; 314 of them had POAG. Among the 314 POAG subjects, 160 of them had HTG and 154 had NTG; 207 (65.9%) were known cases, diagnosed before the start of the study and 107 (34.1%) were previously undiagnosed. The mean age of the 314 participants was 74.2 years (range 49–90 years) and 45% were women. Table 1 summarises the characteristics of the participants.

**Table 1:** Characteristics of the previously known versus previously undiagnosed primary open angle glaucoma participants

Characteristics median (IQR) or %		Previously diagnosed	Previously undiagnosed
n		207	107
Age, years (n*=*314)		72.8 (67.0 to 78.4)	75.4 (70.3 to 81.0)
Sex (n*=*314)	Male	52.2%	61.7%
	Female	47.8%	38.3%
Social class (n*=*314)	Professional/managerial	48.3%	53.3%
	Skilled (manual/non-manual)	41.6%	25.7%
	Semi-skilled/unskilled	10.1%	21.0%
Education (n*=*314)	No qualifications	30.4%	29.9%
	O levels	7.3%	10.3%
	A levels	48.8%	48.6%
	Degree	13.5%	11.2%
Currently employed? (n*=*310)	No	89.3%	79.8%
	Yes	10.7%	20.2%
How often do you not have enough money for basics? (n*=*293)	Never	63.9%	65.7%
	Seldom/sometimes	36.1%	34.3%
POAG type (n*=*314)	HTG	69.1%	15.9%
	NTG	30.9%	84.1%
IOPg (mm Hg) (n*=*307)		16.8 (16.7 to 18.2)	19.0 (17.6 to 19.4)
Pretreatment IOP (mm Hg) (n*=*212)*		26.7.0 (25.9 to 27.5)	18.3 (17.6 to 19.1)
Axial length (mm) (n*=*286)		23.9 (23.2 to 25.1)	23.9 (23.1 to 24.8)
LogMAR visual acuity (n*=*312)		0.20 (0.16 to 0.24)	0.08 (0.0 to 0.26)
Disc photo CDR (n*=*249)		0.55 (0.54 to 0.57)	0.50 (0.48 to 0.53)
CDR asymmetry (n*=*210)		0.06 (0.02 to 0.11)	0.07 (0.04 to 0.11)
Visual field mean deviation		−4.79 (−7.72 to 5.57)	−3.40 (−4.63 to –3.31)
Central corneal thickness (μm)		535 (532 to 543)	544 (536 to 551)
Family history of glaucoma (n*=*269)	No	64.0%	77.7%
	Yes	36.0%	22.3%
Wears glasses/contact lenses? (n*=*308)	No	2.5%	0.96%
	Yes	97.6%	99.0%
Do you have any problems with eyesight? (n=298)	No	11.5%	75.5%
	Yes	88.5%	24.5%
Pseudophakic in either eye (n=314)	No	62.3%	85.1%
	Yes	37.7%	15.0%
Absolute refractive error (Dioptres) (n=309)		1.81 (0.75 to 2.63)	1.25 (0.63 to 2.25)
Systolic BP (mm Hg) (n=314)		137.5 (135.5 to 140.0)	140 (135.8 to 141.8)
Diastolic BP (mm Hg) (n=314)		76.5 (76.0 to 79.6)	79.0 (76.4 to 80.4)
BMI (kg/m^2^) (n=313)		26.0 (26.0 to 27.0)	26.6 (26.1 to 27.6)
Diabetes	No	97.1%	98.1%
	Yes	2.9%	1.9%
Self-reported health status (n=312)	Excellent/very good	37.4%	33.0%
	Good	44.2%	47.2%
	Fair	16.5%	15.1%
	Poor	1.9%	4.7%

Median (95% CI) shown for continuous variables.

*Unimputed data for pretreatment IOP.

BMI, body mass index; BP, blood pressure; HTG, high tension glaucoma; IOPg, Goldmann-correlated intraocular pressure; NTG, normal tension glaucoma.

Table 2 shows the univariable logistic regression results comparing known POAG to previously undiagnosed POAG cases (0=known POAG, 1=previously undiagnosed POAG). The factors associated with previously undiagnosed POAG in the univariable regression were: younger age, being currently employed, having NTG rather than HTG, lower pretreatment IOP, smaller CDR, visual field mean deviation, negative family history of glaucoma, reporting no problems with eyesight, being phakic rather than pseudophakic in either eye and higher absolute refractive error.

**Table 2:** Univariable logistic regression of previously diagnosed versus previously undiagnosed primary open angle glaucoma (0=diagnosed 1=undiagnosed)

Characteristics		OR (95% CI)	P value
Age, years		0.96 (0.93 to 0.99)	0.008
Sex	Male	1.00	
	Female	0.68 (0.42 to 1.09)	0.15
Social class	Professional/managerial	1.00	
	Skilled (manual/non-manual)	0.57 (0.33 to 0.98)	0.04
	Semi-skilled/unskilled	1.84 (0.93 to 3.63)	0.08
Education	No qualifications	1.00	
	O levels	1.44 (0.59 to 3.50)	0.42
	A levels	1.01 (0.59 to 1.74)	0.96
	Degree	0.84 (0.38 to 1.88)	0.68
Currently employed?	No	1.00	
	Yes	2.12 (1.10 to 4.06)	0.02
How often do you not have enough money for basics?	Never	1.00	
	Seldom/sometimes	0.93 (0.56 to 1.54)	0.77
POAG type	HTG	1.00	
	NTG	11.8 (6.52 to 21.5)	<0.0001
IOPg (mm Hg)		1.04 (0.99 to 1.09)	0.08
Pretreatment IOP (mm Hg)*		0.75 (0.69 to 0.82)	<0.0001
Axial length (mm)		1.12 (0.94 to 1.34)	0.21
LogMAR visual acuity		0.86 (0.39 to 1.89)	0.70
Disc photo CDR ×10		0.69 (0.55 to 0.87)	0.02
CDR asymmetry ×10		0.74 (0.48 to 1.15)	0.19
Visual field (MD)		1.14 (1.06 to 1.22)	p<0.0001
Central corneal thickness (μm)		1.01 (0.99 to 1.01)	0.1
Family history of glaucoma	No	1.00	
	Yes	0.51 (0.29 to 0.91)	0.02
Wears glasses/contact	No	1.00	
lenses?	Yes	2.59 (0.30 to 22.4)	0.39
Do you have any problems	No	1.00	
with eyesight?	Yes	0.04 (0.02 to 0.08)	<0.0001
Pseudophakic in either eye	No	1.00	
	Yes	0.29 (0.16 to 0.53)	<0.0001
Absolute refractive error (Dioptres)		1.16 (1.01 to 1.33)	0.03
Systolic BP (mm Hg)		1.00 (0.99 to 1.02)	0.60
Diastolic BP (mm Hg)		1.01 (0.99 to 1.04)	0.32
BMI (kg/m^2^)		1.02 (0.96 to 1.08)	0.50
Diabetes	No	1.00	0.59
	Yes	0.64 (0.13 to 3.22)	
Self-reported health status	Excellent/very good	1.00	
	Good	1.21 (0.71 to 2.05)	0.48
	Fair	1.04 (0.51 to 2.12)	0.93
	Poor	2.75 (0.70 to 10.9)	0.15

*Imputed data.

CDR, cup/disc ratio; HTG, high tension glaucoma; IOP, intraocular pressure; NTG, normal tension glaucoma; POAG, primary open angle glaucoma.

In the final multivariable model (table 3), subjects with previously undiagnosed POAG compared with those with a known diagnosis were more likely to have: a lower pretreatment IOP (OR 0.71/mm Hg, 95% CI 0.63 to 0.80, p<0.0001), and to have reported no problems with their eyesight (OR 0.03, 95% CI 0.01 to 0.69, p<0.0001). The type of glaucoma was strongly associated with the pretreatment IOP, with the IOP being significantly lower among NTG than HTG patients (OR 0.63/mm Hg, 95% CI 0.55 to 0.72, p<0.0001).

**Table 3:** Multivariable regression of previously diagnosed versus previously undiagnosed primary open angle glaucoma (0=diagnosed 1=undiagnosed)

Characteristics		OR (95% CI)	P value
Pretreatment IOP (mm Hg)*		0.71 (0.63 to 0.80)	<0.0001
Do you have any problems with eyesight?	No	1.00	<0.0001
	Yes	0.03 (0.01 to 0.69)	

*Imputed data.

IOP, intraocular pressure.

Factors removed from the model in order were: age (p=0.52), being currently employed (p=0.60), visual fields mean deviation (p=0.40), pseudophakia in either eye (p=0.76), absolute refractive error (p=0.13), family history (p=0.10), CDR (p=0.12) and glaucoma type (p=0.09).

## Discussion

Among the social, economic, systemic and ocular factors examined, the two factors associated with undiagnosed POAG in the community were lower pretreatment IOP and the participants reporting no eyesight problems. The first points to an over-reliance on IOP level to exclude glaucoma in the community, leading to patients with lower IOP to be missed. The National Institute for Health and Clinical Excellence guidelines in the UK recommend a referral to the Hospital Eye Service if IOP is greater than 24 mm Hg in the absence of other risk factors, as ocular hypertension on its own may warrant treatment.^9^ Nevertheless, IOP has been shown to be an ineffective tool for glaucoma case finding in the EPIC-Norfolk Eye Study, and no single IOP level provides both high sensitivity and specificity in glaucoma diagnosis.^8^ This study demonstrates that it is easy for eye care providers to be reassured by an IOP level <24 mm Hg, while other features of glaucoma are missed. It must be stressed therefore that among patients with non-elevated IOP, care should be taken to examine the optic disc carefully and with supportive disc imaging and visual field testing to improve the chances of identifying suspicious disc features.

In this study, other features of severity of glaucoma such as vertical CDR and visual field mean deviation were not associated with missed OAG cases. It could be because CDR does not adequately capture features of a glaucomatous disc, and visual fields may not be done routinely at the optician. Even with advanced field defects, many patients with glaucoma are asymptomatic, so field defects will not necessarily provide a reason to visit the optician.

Several factors were examined that could reflect a participant’s access or likelihood to seek eye care. An important limitation of the methodology is that specific questions about the participants’ history of visits to eye care professionals were not collected prospectively, and asking the questions retrospectively would be prone to recall bias and significant inaccuracies. Hence, there was the need to use proxy factors, such as absolute refractive error, glasses use, being pseudophakic and self-reported visual problems, which require careful interpretation. The strongest factor associated with undiagnosed POAG was answering no to the question ‘do you have problems with your eyesight’, while worse acuity of either eye was not related. A possible interpretation of this finding is that it was the self-perception of good eyesight, and by implication, a lesser likelihood to visit an optometrist, rather than actual visual function, that led the POAG to be undiagnosed. Nevertheless, the participants with existing glaucoma might have perceived their eyesight as being worse than those with undiagnosed glaucoma as a recall bias, as they had a known eye condition, or the use of eyedrops or having had eye surgery diminished their visual function. Wearing glasses or contact lenses was not a significant factor, most likely because 97% of the cohort wore glasses, and it was not effective in discriminating those with previously diagnosed and undiagnosed POAG.

Published studies have explored the question whether visits to eye care professionals is important in facilitating the discovery of glaucoma. The Thessaloniki Study found that previously undiagnosed patients were more likely to not have seen an eye doctor in the past year.^4^ Similarly, both the Barbados Eye Study^3^ and the Melbourne Visual Impairment Project^5^ reported that previously undiagnosed patients sought eye care less frequently in the past year, with the source of eye care more likely to be an optometrist rather than an ophthalmologist.^3 5^ However, these findings are potentially confounded by the fact that patients with diagnosed glaucoma would already be under the care of an ophthalmologist, so a prospective study is required to adequately answer that question.

Currently in the UK, POAG is diagnosed by opportunistic case finding, relying on patients presenting to an optometrist for an eye test, and referral made to the Hospital Eye Service under the National Health Service if glaucoma is suspected. Those most at risk of glaucoma—aged >60 and those aged >40 with a positive family history of glaucoma in a first degree relative—can get the optician’s eye test for free, and so there is no financial barrier to the diagnosis of glaucoma among those at risk. In EPIC-Norfolk, a greater proportion of all glaucoma cases (67%) were previously diagnosed, compared with the usual 50% reported in most western population studies. There are a few potential explanations. First, due to the method of recruitment and the low proportion of retained participants from the original cohort, the study population was not representative of the general population of Norfolk and UK. They were older, with an over-representation of those above age 60, the age from which eye tests are free at the optician, therefore biasing the likelihood of them visiting an optician. Another explanation could be recruitment bias, as health conscious individuals who were more likely to visit their optician were also more likely to participate in this study. It might also reflect more effective healthcare provision in Norfolk. However, one cannot discount the possibility that some participants who were known patients with glaucoma were motivated to take part by an enthusiasm to have additional eye testing, causing response bias.

In conclusion, the most important healthcare implication from this analysis is to avoid being falsely reassured by a lower level of IOP in glaucoma case finding. There is also a suggestion that raising public awareness of glaucoma and encouraging regular eye tests at the optician can help reduce undiagnosed glaucoma.

## Data Availability

Data are available upon reasonable request. All data relevant to the study are included in the article or uploaded as supplementary information. All data relevant to the study are included in the article or uploaded as supplementary information. Data are available upon reasonable request
